# Visualization of a Bruton’s Tyrosine Kinase Inhibitor Using Fluorescence and Raman Microscopy

**DOI:** 10.1021/acs.analchem.5c04965

**Published:** 2026-05-04

**Authors:** Andrew S. Merchant, William J. Tipping, Duncan Graham, Karen Faulds

**Affiliations:** Bionanotechnology, Department of Pure and Applied Chemistry, Technology and Innovation Centre, 3527 The University of Strathclyde, 99 George Street, Glasgow G1 1XL, U.K.

## Abstract

Cellular imaging is important in understanding drug pharmacokinetics and dynamics. As such, it is crucial that the drug is unmodified when performing these studies, to neither inhibit nor change its action and properties. Historically, fluorescence microscopy has been used for drug imaging due to its high sensitivity and ease of use, but bulky fluorescent tags have the potential to cause off-target effects and result in a change in the pharmacokinetic properties. The use and development of small optical tags are therefore attractive, as cellular systems can be probed with minimal perturbation to the cellular environment and the native kinetics of a drug. Bio-orthogonal Raman imaging makes use of molecular vibrations that are seldom observed in nature to determine spatial localization. Spontaneous Raman scattering can be used to achieve minimally labeled drug localization but is relatively slow and has a low spatial resolution when compared to fluorescence microscopy. Faster image acquisition and higher spatial resolution can be achieved by using stimulated Raman scattering (SRS), a powerful technique that is often used for native cellular imaging. The use of either intrinsically bio-orthogonal drugs or those with a small tag added allows Raman scattering to be used as a companion imaging diagnostic tool. This work assesses the localization of a covalently binding inhibitor of Bruton’s tyrosine kinase, ibrutinib, using fluorescently labeled and bio-orthogonally Raman labeled analogues as companion diagnostic tools. Localization of these analogues was determined using fluorescence and Raman microscopies, and inhibitor retention was proportional to the expression of the kinase. Significant retention of the fluorescent analogue was observed independent of kinase expression, indicating significant nonspecific binding. Drug-induced effects were also explored using spectral phasor analysis of hyperspectral SRS data to assess lipid metabolism, where BTK inhibition was shown to cause an increase in the lipid content and change in the lipid type, which was proportional to kinase expression. This work showcases the advantages of Raman scattering techniques over fluorescence as companion imaging diagnostic tool and as a method of assessing phenotypic lipid shifts upon treatment with an anticancer drug.

## Graphical abstract

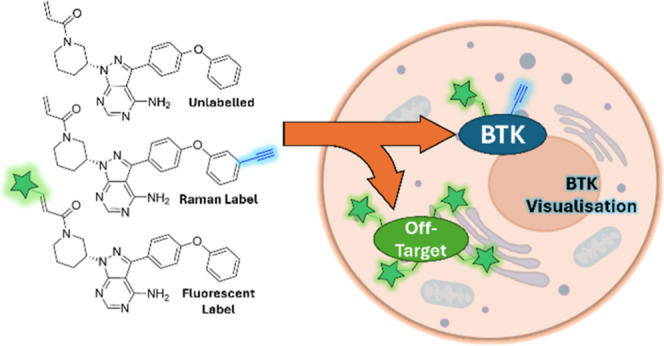

## Introduction

Bruton’s tyrosine kinase (BTK) is a nonreceptor bound tyrosine kinase found in the cytoplasm of a wide range of epithelial cells and plays a pivotal role in the proliferation of B cells.^1^ Dysfunction and dysregulation of BTK may result in malignancies such as cancer,^1^ and a wide range of small molecule BTK inhibitors have been developed to arrest the effects of dysregulated BTK.^2^ First generation, covalent inhibitor, ibrutinib, was developed as an ATP mimic with an acrylamide warhead to irreversibly bind to C481 in the ATP binding domain of BTK.^3^ Since ibrutinib, subsequent generations of BTK inhibitors have been developed with superior selectivity, to counteract the significant off-target effects associated with ibrutinib across multiple tyrosine kinases, serine/threonine kinases, and growth factor receptors.^4^

Understanding BTK inhibitor binding has previously been established through fluorescent probes as companion imaging diagnostics tools,^5^ which can assist in providing information on drug localization, metabolism, and fate.^5–7^ One such example is PCI-33380,^8^ an ibrutinib analogue containing a BODIPY tag on the acrylamide warhead. However, this analogue resulted in a 100-fold decrease in potency compared to the parent drug (IC_50_ of ∼200 nM compared to ∼2 nM).^8^ In addition to PCI-33380, multiple fluorescent probes for ibrutinib have been developed^9^ to augment size and fluorescent properties, but still these probes feature large linkers and bulky fluorescent groups that may add upward of 400 Da to the native drug, potentially leading to nonspecific binding and a decrease in efficacy. As such, there is a great need for the development of smaller optical tags which impart less structural perturbation and ultimately the ability to perform label-free imaging of BTK inhibitor interactions *in vitro*.

Within the past decade, the addition of small, Raman-based, bio-orthogonal optical tags to molecules has gained increasing interest.^10^ Recently, Raman spectroscopy has been used extensively for imaging small molecules using bio-orthogonal groups with vibrational peaks that are not observed in cellular spectra.^11^ The region in which no endogenous cellular peaks are observed is known as the “cell-silent region” (∼1800–2800 cm^–1^). Functional groups which give vibrational peaks in this region include triple bond stretches^12^ and C–D stretches,^13^ as well as B–H^14^ and azide stretches,^15^ which can be utilized for bio-orthogonal imaging. Fingerprint Raman spectroscopy can also be used to visualize characteristic drug peaks^16^ or track bond formation.^17^ Coherent Raman techniques, such as stimulated Raman scattering (SRS), can give rapid, high resolution, real-time imaging of drug localization within cells, with superior speed and spatial resolution compared to spontaneous Raman scattering. This has been extensively documented for multiple small molecules, both with intrinsic bio-orthogonal groups,^10,18,19^ with a small bio-orthogonal tag added,^20,21^ or by using the fingerprint spectrum of the drug.^16^

Raman spectroscopy can also be used to track changes in lipids and proteins, which is especially useful in cancer research, as lipid metabolism is often dysregulated.^22^ Changes in lipid-to-protein ratio can be observed in Raman spectra, which can often be more easily visualized using multivariate analysis.^23–25^ The dysregulation of lipid metabolism can also be observed in hyperspectral SRS (hsSRS) data and visualized using spectral phasor analysis (SPA).^23,24^ SPA groups hsSRS pixels based on their spectral similarity and has been used to assess phenotypic variance in cells in several studies.^24,26–28^

In this work, a panel of mammalian cancer cells was used to assess the binding and distribution of ibrutinib, using a selection of Raman- and fluorescence-based probes with various microscopic techniques. A comparison of the current “gold standard” of a fluorescent analogue to the bio-orthogonal equivalent using SRS was initially performed, along with an assessment of lipid content between drug treated and control cells using SPA. The greater spectral information received from spontaneous Raman scattering in comparison to SRS was also showcased using the distribution of the same bio-orthogonal analogue, as well as probing the low wavenumber region of a spontaneous Raman spectrum to allow for identification of changes in lipid identity and content in response to drug treatment, but with the greater spectral information allowing for more detailed assessment. Together, this study shows the use of Raman-based microscopic techniques in probing drug distribution and drug-induced effects *in cellulo* compared with some fluorescence-based techniques.

## Materials and Methods

### Reagents and Chemicals

Ibrutinib and ibrutinib-yne (PF-06658607) were purchased from Merck, and ibrutinib-FL (PCI-33380) was purchased from MedChemExpress. Solids were used as supplied or prepared as 10 mM stock solutions in anhydrous dimethyl sulfoxide (DMSO).

### Cell Culture

K562 cells (ATCC CCL-243) and HeLa cells (ATCC CCL-2) were obtained from American Type Culture Collection. K562 cells were cultured in Rosewell Park Memorial Institute medium (RPMI 1640; GIBCO, Fisher Scientific) supplemented with 10% fetal bovine serum (FBS, GIBCO, Fischer Scientific), 1% penicillin/streptomycin (GIBCO, 10,000 U mL^–1^, Fisher Scientific), and 1% Amphotericin B (GIBCO, 250 μg mL^–1^, Fisher Scientific). HeLa cells were cultured in Dulbecco’s Modified Eagle’s medium (DMEM low glucose, 1 g L^–1^) supplemented with 10% FBS (GIBCO, Fischer Scientific), 1% penicillin/streptomycin (GIBCO, 10,000 U mL^–1^, Fisher Scientific), and 1% Amphotericin B (GIBCO, 250 μg mL^–1^, Fisher Scientific). All cells were maintained at 37 °C and 5% CO_2_ in a humidified incubator and were routinely subcultured at *ca.* 80% confluency.

### Raman Microscopy

All Raman spectra were acquired on a Renishaw inVia Raman microscope equipped with a 532 nm Nd/YAG laser providing a maximum output of 50 mW (38.3 mW on sample) and using an 1800 lines per mm grating, and a 785 nm diode laser providing a maximum output of 300 mW (142 mW on sample) and using a 1200 lines per mm grating. Prior to spectral acquisition, the instrument was calibrated using an internal silicon standard at 520.5 cm^–1^.

### Solid and Solution Phase Analysis

Aliquots of solid or solution were spotted onto a CaF_2_ disc (Crystran, UK) and analyzed using 785 or 532 nm laser excitation using a 20× air objective lens (Leica Microsystems, N PLAN EPI, N.A. 0.4).

### Live-Cell Analysis

Live HeLa cells were plated onto CaF_2_ discs (Crystran, UK) in a 6-well plate in their respective media at a concentration of 0.5 million cells per mL. The cells were cultured for 24 h before treatment with BTK inhibitor or DMSO (0.1% v/v control) for the indicated time points and concentrations. The media was removed, and discs were washed with 2 mL of phosphate buffered saline (PBS, pH 7.4, Oxoid) 3×. Discs were transferred to a glass-bottomed culture dish (Ibidi) and submerged in 3 mL of PBS. Cells were analyzed using 785 or 532 nm laser excitation, using a 60× water immersion lens (Nikon, NIR Apo water immersion, N.A. 1.0), 1 μm step size in *x* and *y*, 0.5 s, 50% laser power (ca. 71 mW or 18 mW) with a spectral center of 2700 cm^–1^.

Live K562 cells were placed in T25 flasks at a concentration of 0.2 million per mL. The flasks were then treated with BTK inhibitor or DMSO (0.1% v/v control) for the indicated time points and concentrations. Cells were then centrifuged (1000 rpm, 5 min) and concentrated to *ca.* 2 million per mL by pipetting off extraneous media. Cells were then washed in 5 mL of PBS, centrifuged (1000 rpm, 5 min), and concentrated to *ca.* 2 million per mL by pipetting off extraneous PBS. This washing was repeated for a total of three washes. A drop of the concentrated cell solution was placed and spread onto a CaF_2_ disc (Crystran, UK) and analyzed using 532 nm laser excitation using a 20× or 50× (Leica, N PLAN EPI, N.A. 0.75) air objective lens or 60× water immersion lens as above.

### Fluorescence Microscopy

Fluorescence images were acquired using 405 and 488 nm lasers coupled into an inverted laser-scanning microscope (Leica TCS SP8, Leica Microsystems). Fluorescent images were acquired using a 63× objective (HC PL APO 63×, N.A. 1.40 oil immersion lens) with a 48 μs pixel dwell time over a 512 × 512 px frame. Images were acquired at 10 Hz with excitation at a 12-bit image depth. Colonies of cells were selected using DAPI (5 μM, λ_ex_ = 405 nm, λ_em_ = 410–500 nm) as a marker for nuclei.

### Live-Cell Fluorescence Imaging

Live K562 cells were placed in a T25 flask at a concentration of 0.2 million cells per mL. The flask was then treated with ibrutinib-FL (500 nM, 4 h, λ_ex_ 458 nm, λ_em_ 530–630 nm). Cells were then centrifuged (1000 rpm, 5 min) and concentrated to ca. 2 million per mL by pipetting off extraneous media. Cells were then washed with PBS (2 × 5 mL), centrifuged between washes (1000 rpm, 5 min), and concentrated to *ca.* 2 million cells per mL by pipetting off extraneous PBS. The cell suspension was counterstained with DAPI (2 μM), and a drop was placed onto a microscope slide, covered by a high precision glass coverslip (no. 1.5H thickness, 22 × 22 mm, Thorlabs) and sealed with nail polish. HeLa cells were plated onto high precision glass coverslips (#1.5H thickness, 22 × 22 mm, Thorlabs) in a 6-well plate in their respective media at a concentration of 0.5 million per mL and treated with ibrutinib-FL (500 nM, 4 h, λ_ex_ 458 nm, λ_em_ 530–630 nm) and counterstained with DAPI. The slide was washed with PBS inverted, mounted with a boundary of PBS, and sealed with nail polish, and imaged as previously described.

### Immunofluorescence

HeLa cells were plated onto high precision glass coverslips (no. 1.5H thickness, 22 × 22 mm, Thorlabs) in a 6-well plate in DMEM at a concentration of 0.5 million per mL, and K562 cells were placed in a T25 flask at a concentration of 0.2 million cells per mL. Cells were either left as is or treated with ibrutinib (10 or 50 μM). After 24 h, cells were fixed using 4% PFA for 15 min at room temperature before rinsing 3× with PBS for 5 min each. Cells were then blocked using a 5% BSA (ThermoFisher) solution in PBS containing 0.3% Triton X-100 (Merck). Monoclonal mouse BTK antibody (Thermofisher, MA5-15,337) was diluted 1:500 in a solution containing 1% BSA solution in PBS containing 0.3% Triton X-100, and cells were treated with this antibody solution overnight at 5 °C. Cells were then rinsed 3× with PBS for 5 min each, before treating with AlexaFluor 633 conjugated secondary polyclonal mouse antibody (Thermofisher, λ_ex_ =633 nm, λ_em_ =640–700 nm) diluted 1:1000 in 1% BSA solution in PBS containing 0.3% Triton X-100 for 2 h at 4 °C. Cells were then rinsed 3× with PBS for 5 min each, counterstained with DAPI as previously, and mounted and imaged as previously described.

### SRS Microscopy

An integrated laser system (picoEmerald S, Applied Physics & Electronics, Inc.) was used to produce two synchronized laser beams at 80 MHz repetition rate. A fundamental Stokes beam (1031.4 nm, 2 ps pulse width) was intensity modulated by an electro-optic-modulator (EoM) with >90% modulation depth, and a tunable pump beam (700–960 nm, 2 ps pulse width, <1 nm (10 cm^–1^) spectral bandwidth) was produced by a built-in optical parametric oscillator. The pump and Stokes beams were spatially and temporally overlapped using two dichroic mirrors and a delay stage inside the laser system and coupled into an inverted laser-scanning microscope (Leica TCS SP8, Leica Microsystems) with optimized near-IR throughput. SRS images were acquired using 40× objective (HC PL IRAPO 40×, N.A. 1.10 water immersion lens) with a 9.75–48 μs pixel dwell time over a 512 px × 512 px frame. The Stokes beam was modulated with a 20 MHz EoM. Forward scattered light was collected by a S1 N.A. 1.4 oil immersion condenser lens (Leica Microsystems). Images were acquired at 12 bit image depth. The laser powers measured after the objective lens were in the range 10–30 mW for the pump beam only, 10–50 mW for the Stokes beam only, and 20–70 mW (pump and Stokes beams).

### Live-Cell SRS Imaging

HeLa cells were plated onto high precision glass coverslips (no. 1.5H thickness, 22 × 22 mm, Thorlabs) in a 6-well plate in DMEM at a concentration of 0.5 million per mL, and K562 cells were placed in a T25 flask at a concentration of 0.2 million per mL. Cells were treated and slides prepared as described previously. The slide was mounted onto the multiphoton instrument, and a colony of cells selected using 2930 cm^–1^ as a reference for proteins. Images were acquired at 10 Hz at 2930, 2851, 2111, and 2180 cm^–1^.

### hsSRS Scans

HeLa cells were plated onto high precision glass coverslips (no. 1.5H thickness, 22 × 22 mm, Thorlabs) in a 6-well plate in DMEM at a concentration of 0.5 million per mL, and K562 cells were placed in a T25 flask at a concentration of 0.2 million per mL. Cells were treated and mounted as described previously. The slide was mounted onto the multiphoton instrument, and a colony of cells selected using 2930 cm^–1^ as a reference for proteins. 40 scans at 50 Hz of this colony of cells were performed between ∼3050 and 2800 cm^–1^, with a wavelength difference between each scan of 0.4 nm.

### Data Processing

#### Raman Maps

Raman maps were created as described in ref 29 in brief:

*Renishaw Wire 4.1* was used to perform basic preprocessing steps using the built-in functions in the software. Cosmic rays were removed, followed by noise filtering and baseline subtraction.

Custom MATLAB scripts were then used to perform further analysis. Cell regions were selected based on the total spectral intensity for the map, and all associated spectra were extracted for comparison between conditions. False color images for the cell regions were created based on appropriate ratios

$$Lipid/Protein-\frac{I\left(2851\;cm^{-1}\right)}{I\left(2930\;cm^{-1}\right)+I\left(2851\;cm^{-1}\right)}$$

$$Alkyne/Protein-\frac{I\left(2211\;cm^{-1}\right)}{I\left(2930\;cm^{-1}\right)+I\left(2211\;cm^{-1}\right)}$$

Mean spectra were also obtained for the cell area.

### Raman Spectra

All Raman spectra were plotted using GraphPad Prism software, with spectra being normalized to the most intense peak. Peak area and shifts were assigned using the integrate and pick peak functions on Origin2018.

### SRS and Fluorescence Images

False color assignments, scale bars, and image overlays were added to images using ImageJ software. Consistent brightness and contrast settings were used when comparing image data sets. Images of ibrutinib-yne distribution are presented by subtracting the background signal from the on-resonant signal (2111–2180 cm^–1^) using the Image Calculator function available on ImageJ. Images of ibrutinib-FL and BTK antibody distribution are presented by merging channels showing localization of DAPI and ibrutinib-FL or AlexaFluor633.

### hsSRS Scans

SRS scans were imported into ImageJ software. An average intensity projection of the scan was performed, and the threshold was adjusted to create a mask of cellular regions, removing the background. The scan was then multiplied by the mask using the *Image Calculator* function on ImageJ. SPA was then performed using the spectral phasor plugin^23^ to discriminate different cellular components. Sections of this phasor were then manually selected using the polygon selections tool, and these were converted to images using the *Phasor to Image* plugin.^23^ False color assignments and scale bars were added to these different cellular components and merged into one image using the *Merge* tool on ImageJ.

## Results and Discussion

Ibrutinib (Figure 1A) and two companion imaging diagnostic probes were chosen to study and compare uptake and retention in vitro; PF-06658607—an alkynyl derivative (Figure 1B) known herein as ibrutinib-yne, and PCI-33380—a fluorescent BODIPY derivative (Figure 1C) known herein as ibrutinib-FL. Ibrutinib-yne was produced by Pfizer to allow for further labeling using click chemistry.^30^ This not only allows for the attachment of azido-fluorophores distal to the warhead but also facilitates bio-orthogonal Raman imaging. The addition of an alkyne only increases the molecular weight by 24 Da and is far from the covalent binding site which, in comparison to ibrutinib-FL with the fluorophore attached to the warhead, was proposed to have less effect on the action of the BTK inhibitor in binding to C481 in the ATP binding domain of BTK.^30^ Spontaneous Raman scattering was performed on both ibrutinib and ibrutinib-yne, which produced similar spectra (Figure 1D), especially in the fingerprint region, but a distinct alkyne peak was observed at 2110 cm^–1^ in ibrutinib-yne. Some minor differences were observed between the two spectra in the fingerprint region, especially around the aromatic region, with an increase in intensity at 998 cm^–1^ for ibrutinib-yne, relating to ring breathing. The increase is possibly due to electronic resonance of the π-system incorporating the alkyne group. Other similarities include peaks around 600 cm^–1^ relating to the acrylamide, peaks around 1450 cm^–1^ relating to aliphatic CH_2_, and peaks around 1550 cm^–1^ related to the heterocyclic rings, which are all unaffected by the modification. The presence of the sharp, discrete alkyne peak at 2110 cm^–1^ allows ibrutinib-yne to be used as a small, optically tagged analogue of the BTK inhibitor ibrutinib allowing for its localization to be determined in vitro using Raman scattering. Due to the strong fluorescence of ibrutinib-FL, a Raman spectrum was not obtained.

**Figure 1:**
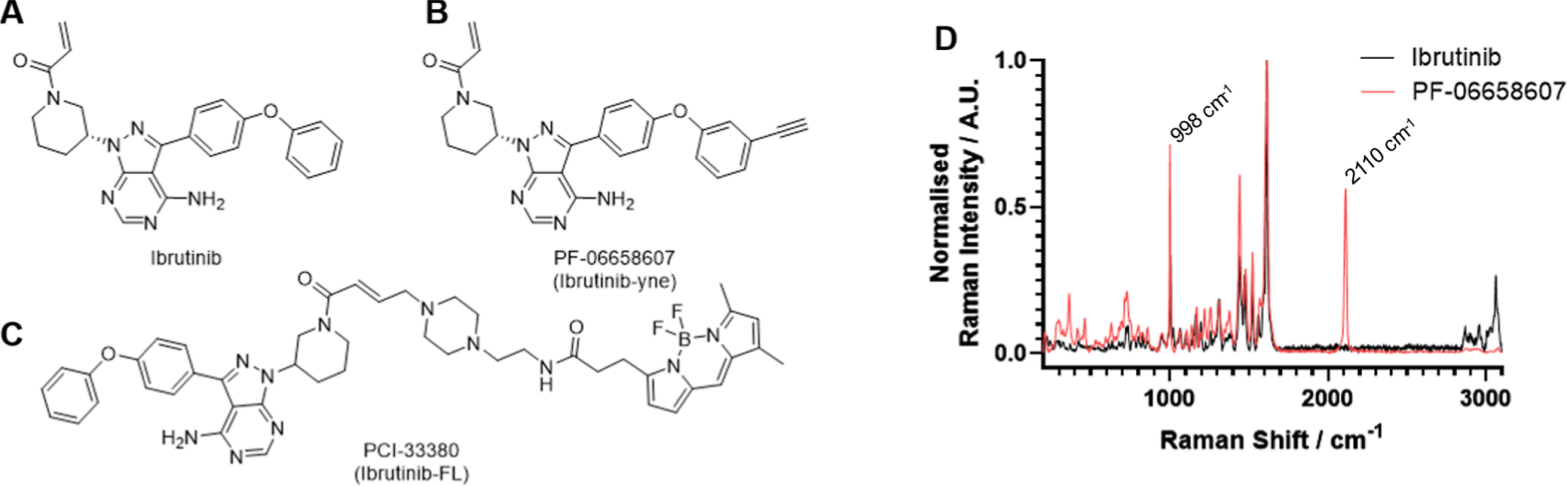
Structure and spontaneous Raman analysis of ibrutinib and companion diagnostic probes. Structure of (A) ibrutinib and (B) ibrutinib-yne, (C) ibrutinib-FL, and (D) the overlaid solid Raman spectra of ibrutinib (black) and ibrutinib-yne (red). Raman spectra were acquired using 785 nm excitation, 10 s integration time, 71 mW laser power, 20× magnification.

High BTK expressing cell line K562 (chronic myelogenous leukemia)^31^ and low BTK expressing cell line HeLa (ovarian cancer)^31^ were chosen for analysis in this work. Immunofluorescence (IF) confirmed the expression level of BTK in the cell lines, with K562 being the highest, followed by HeLa with the lowest expression (Figures 2A and S1). This agreed with previously reported expression data.^31^ Fluorescent imaging was performed using ibrutinib-FL. Due to the strong fluorescence of the modified BODIPY, ibrutinib-FL was able to be detected at low concentrations (<1 μM), but it had a markedly lowered IC_50_ against BTK (200 nM *vs* 2 nM from ibrutinib).^8^ K562 and HeLa cells were treated with ibrutinib-FL (500 nM, 4 h), and after a 30 min wash, significant fluorescence signal was observed in both cell types (Figures 2B and S2A), with HeLa cells having comparable intensity to K562 cells, contradicting the previous IF experiments. A 24 h wash gave a lowered fluorescent signal in both K562 and HeLa cells (Figures 2B and S2A), which may indicate significant nonspecific binding due to the significant increase in hydrophobicity attributed to the modified BODIPY group. A larger decrease in the fluorescence signal was observed in HeLa compared to K562 following washing (Figure 2B), indicating that more of the probe had irreversibly bound to BTK in K562 cells, agreeing with the IF results. A comparative experiment was performed using ibrutinib-yne (Figures 2C and S2B), and no lowering of the SRS intensity of the alkyne was observed after 24 h of washing, with significant differences observed between K562 and HeLa cells. This indicates there is little to no nonspecific binding from this minimally tagged analogue. The SRS intensities show an approximately 2-fold decrease from K562 to HeLa, which is a much less significant decrease than the BTK expression IF data (Figure 2A, ∼90% decrease). The fluorescence signal also shows an approximately 2-fold decrease after a 30 min wash, and a 5-fold decrease after a 24 h wash, which was also less than expected in comparison to BTK expression between the two cell lines. With SRS, this may be due to a higher level of baseline that is not removed with the off-resonance images. To further investigate this, Figure S3 highlights that although off-resonance background is higher compared to K562 cells, it is uniform across the whole image and therefore not due to drug localization within the cells. Fluorescence microscopy has a higher signal to noise ratio due to its brighter signal and will therefore have a decreased noncell-specific background. Although there was a difference in fold-difference in uptake in both fluorescence and SRS methods, both techniques accurately reflect the trend of a higher uptake of their respective BTK probe in K562 cells relative to HeLa cells. These data highlight the benefit of using a Raman tagged companion imaging diagnostic, as they have minimal perturbation on the normal binding and retention of the drug, unlike with bulkier analogues.

**Figure 2:**
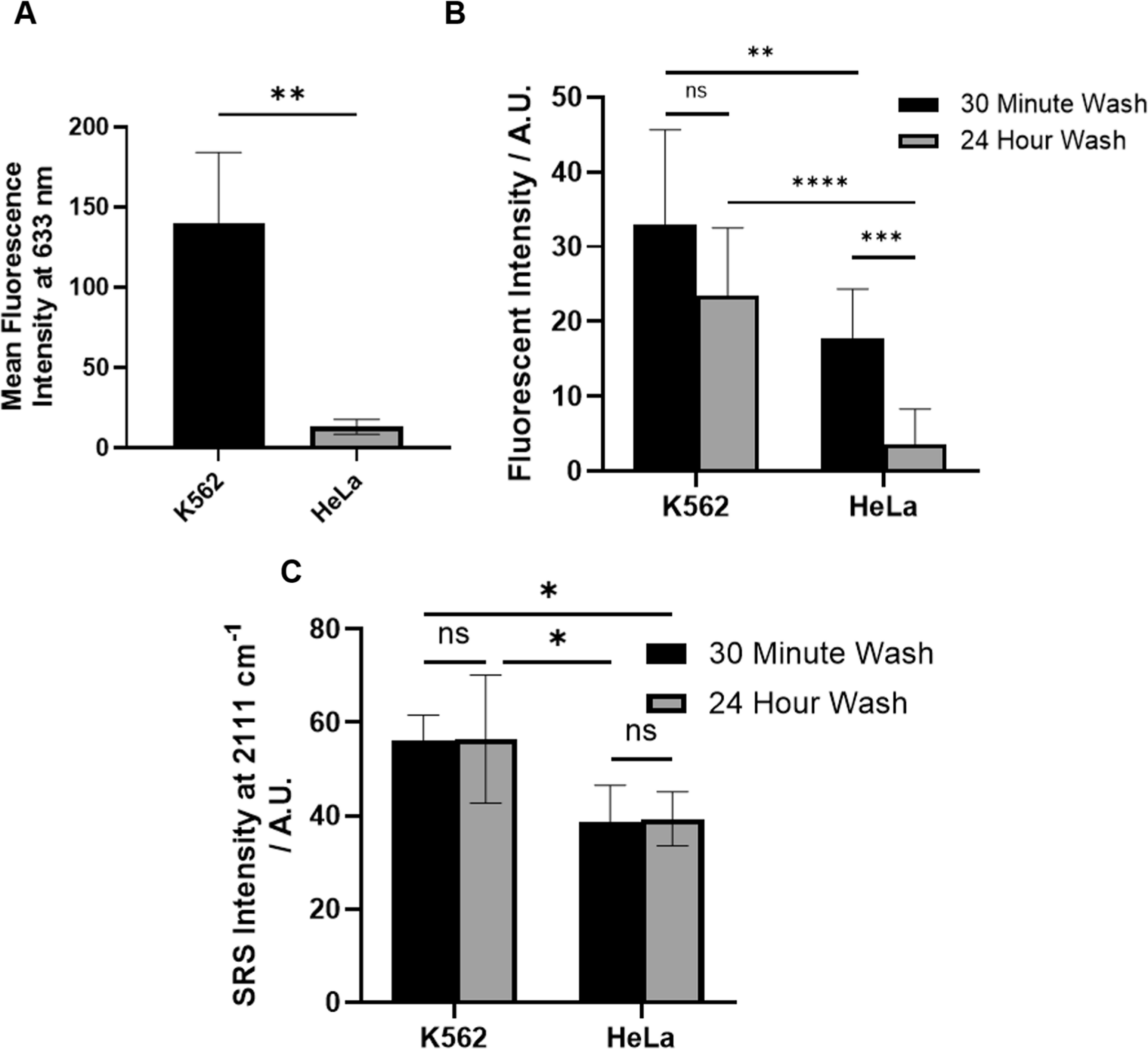
Immunofluorescence analysis of a panel of cell lines and fluorescence and SRS imaging of ibrutinib-FL and ibrutinib-yne in K562 and HeLa cells. (A) Mean fluorescence intensities of an AlexaFluor633 conjugated antibody bound to a BTK antibody in K562 and HeLa cells. (B) Comparisons of fluorescence intensities in both cell lines after 30 min and 24 h washes, and (C) comparisons of background subtracted SRS images at 2111 cm^–1^ in both cell lines after 30 min and 24 h washes. Intensity data represented as mean ± standard deviation (*n* = 9). An unpaired Welch’s *t*-test was performed when comparing fluorescence intensity between cell lines, and a paired *t*-test was performed when comparing between treatments and washes. ns—no significance, *—*P* < 0.05 **—*P* < 0.01, ***—*P* < 0.001, ****—*P* < 0.0001.

As a direct comparison to fluorescence, SRS imaging of K562 cells treated with ibrutinib-yne was employed to evaluate the differences in BTK inhibitor uptake at different concentrations. IC_50_ data for ibrutinib has been shown to be 7.5 μM in K562 cells;^32^ therefore, a treatment concentration of 10 μM for relatively short timeframes was chosen. Uptake of ibrutinib-yne was observed after treatments with 10 μM for both 4 h and 24 h and was localized in the cytoplasm (Figure 3A). The SRS images obtained from K562 and HeLa cells are shown in Figure 3. On treatment with ibrutinib-yne (10 μM, 4 h), K562 cells showed significant blebbing in the SRS images obtained at 2930 cm^–1^ and showed a strong signal at 2111 cm^–1^, relating to the alkyne in ibrutinib-yne (Figure 3A, top). No perturbation of the cell membrane was observed in the protein (2930 cm^–1^) images of HeLa cells, and a minimal alkyne signal was observed on the 2111 cm^–1^ background subtracted image, attributed to background resonance and artifacts from cross-phase modulation^33^ (Figure 3A, bottom). Comparisons of the SRS intensity obtained from off-resonance-subtracted alkyne images between K562 and HeLa cells show a significant decrease in signal in the HeLa cells compared to K562 cells (Figure 3B), as observed with ibrutinib-FL in Figure 2. Figure S3 includes the same alkyne images with increased brightness and contrast to show there was a low intensity, but uniform signal in HeLa across the image acquired from HeLa cells, indicating a noncell-specific background. This work highlights a drawback of fluorescence microscopy, as multiple stains may be required to visualize cellular morphology, whereas SRS can probe cellular architecture and distribution of molecules in a minimally labeled fashion.

**Figure 3:**
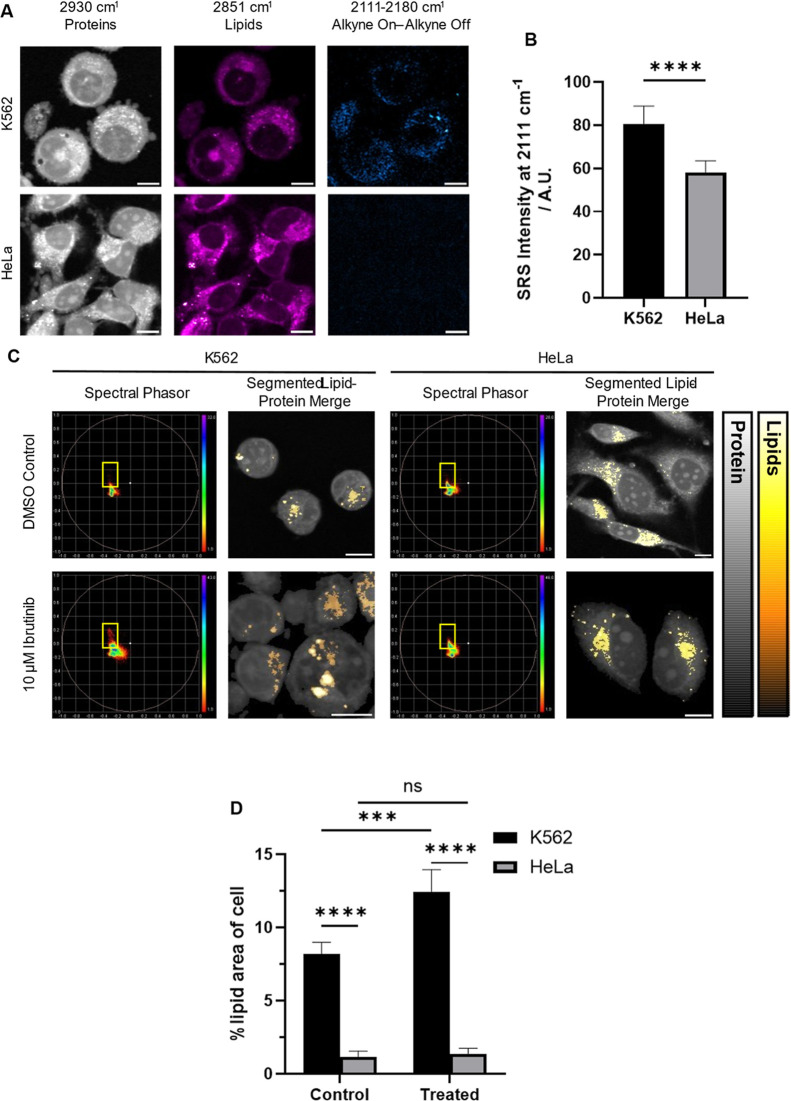
SRS and SPA of K562 and HeLa cells. (A) SRS images at 2930 and 2851 cm^–1^, and an off-resonance subtraction of 2111 cm^–1^ (alkyne)—2180 cm^–1^ (off-resonance) in K562 and HeLa treated with the alkynyl ibrutinib analogue, ibrutinib-yne (10 μM, 4 h) after 30 min wash with PBS, (B) a comparison of SRS intensity of the subtracted images in both cell types, (C) SPA of hyperspectral SRS data between 3050 and 2800 cm^–1^ of K562 and HeLa cells treated with either ibrutinib-yne (10 μM, 4 h) or DMSO (10 μL, 4 h), showing both the phasor plot with lipid regions highlighted by a yellow box, and the distribution of lipids over the average image intensity, and (D) a comparison of lipid area extracted from the phasor plot in relation to the full cell in K562 and HeLa cells, treated as described above. Scale bars: 10 μm. SRS intensity and area data represented as mean ± standard deviation (*n* = 8 and 3 images of ∼20 cells each respectively). An unpaired Welch’s *t*-test was performed on SRS intensity and when comparing area between cell lines, and a paired *t*-test was performed when comparing between treatments and washes, ns: no significance, ***: *p* ≤ 0.001, ****: *p* ≤ 0.0001.

SPA compares spectral similarities of hyperspectral SRS (hsSRS) data and groups pixels based on their spectral similarity.^23^ This allows for the comparison of cytoplasm, lipid, nuclei, and nucleoli area as well as spectra. hsSRS data consists of multiple SRS images ∼7 cm^–1^ apart to allow for highly spatially resolved probing into the Raman spectra of cellular components. SPA was employed on hsSRS data to assess differences in lipid metabolism between the cell type and BTK inhibition. K562 cells showed an increase in lipid content upon treatment as defined by the yellow region on the phasor plot (Figure 3C, left). This may indicate an upregulation in lipids stored compared to normal cellular function due to the formation of lipid-rich regions. By comparing lipid overlays, we found that treated K562 cells showed more concentrated droplets than the control. However, HeLa cells showed little variance in the shape and range of the phasor plot, indicating similar amounts and types of lipids in both samples. The range and distribution of lipid droplet size and localization was consistent in both samples, indicating a less significant response (Figure 3C, right). This was also confirmed by exploring the area of the cell covered by lipid droplets extracted from the phasor plot (Figure 3D). This found a significant increase upon treatment in K562 cells, with no difference in HeLa cells, indicating some difference in lipid metabolism upon treatment in K562 cells. Previous research found that treatment with ibrutinib cased a decrease in lipid droplet accumulation in mantle cell lymphoma,^34^ but in that work, a 5-fold lower concentration of ibrutinib was used compared to this work. Ibrutinib has been shown to inhibit the metabolism of free fatty acids and that lipid metabolism is linked to leukemic survival,^35^ suggesting that lipid accumulation indicates decreased metabolism in response to treatment. hsSRS allows for the unlabeled assessment of endogenous spectral and spatial characteristics, which is not achievable using fluorescence without using multiple stains.

Spontaneous Raman mapping of BTK inhibitor treated cells allows for a greater spectral resolution to be acquired (∼1.4 cm^–1^) compared to SRS (∼7 cm^–1^ due to tuning limitations),^14^ with sharper peaks allowing for better discrimination, at the expense of a lower spatial resolution and speed. Ratiometric spontaneous Raman maps of K562 cells were produced by plotting the ratio of lipids/(lipids + proteins); (2851 cm^–1^/2851 cm^–1^ + 2930 cm^–1^),^29^ and alkyne/(alkyne + proteins); (2111 cm^–1^/2111 cm^–1^ + 2930 cm^–1^) to normalize peak heights to the proteins, showing lipid and alkyne distribution (Figure 4A) from a spectral range of 2000 cm^–1^ to 3100 cm^–1^. These maps were able to show the uptake of ibrutinib-yne at 50 μM, as well as 10 μM within 4 h, although similarly to SRS data, 50 μM treatments caused significant perturbation to the cellular structure. Upon treatment with 50 μM either ibrutinib or ibrutinib-yne, the HeLa cells almost completely detached from the slides, with cells lost on washing. However, each cell line appeared to be able to tolerate 10 μM treatments, with little observable deviation from normal morphology. Ratiometric Raman maps HeLa cells after 10 μM treatment showed significantly lowered accumulation of labeled BTK inhibitor compared to K562, consistent with a lower expression of BTK, shown in Figure 2. This lower accumulation is shown with HeLa (Figure 4B), with the alkyne/protein ratiometric map showing no difference in intensity between cytoplasm and nucleus, compared to the same concentration in K562 which had very good separation between the nucleus and cytoplasm. Average spectra of the ratiometric maps of the cell were obtained, and the area under the curve (AUC) of the alkynyl peak at 2111 cm^–1^ was taken. This showed a trend of ibrutinib-yne uptake that agreed with the IF data in Figure 2; K562 cells had the highest uptake and retention after washing, with HeLa cells having a significantly lowered uptake and retention (Figure 4C). Although spontaneous Raman mapping trades speed and spatial resolution for increased spectral information, the advantage of being able to probe a whole spectral range, with characteristic peaks of a multitude of cellular components being well documented, and able to be distinguished label-free highlights another benefit of Raman spectroscopy over traditional fluorescence imaging. Raman spectroscopy also partially addresses an issue of multiplexing, as ranges of excitation lasers and emission detectors are not required, lowering or in some cases removing crosstalk between endogenous, unlabeled, and labeled molecules.

**Figure 4:**
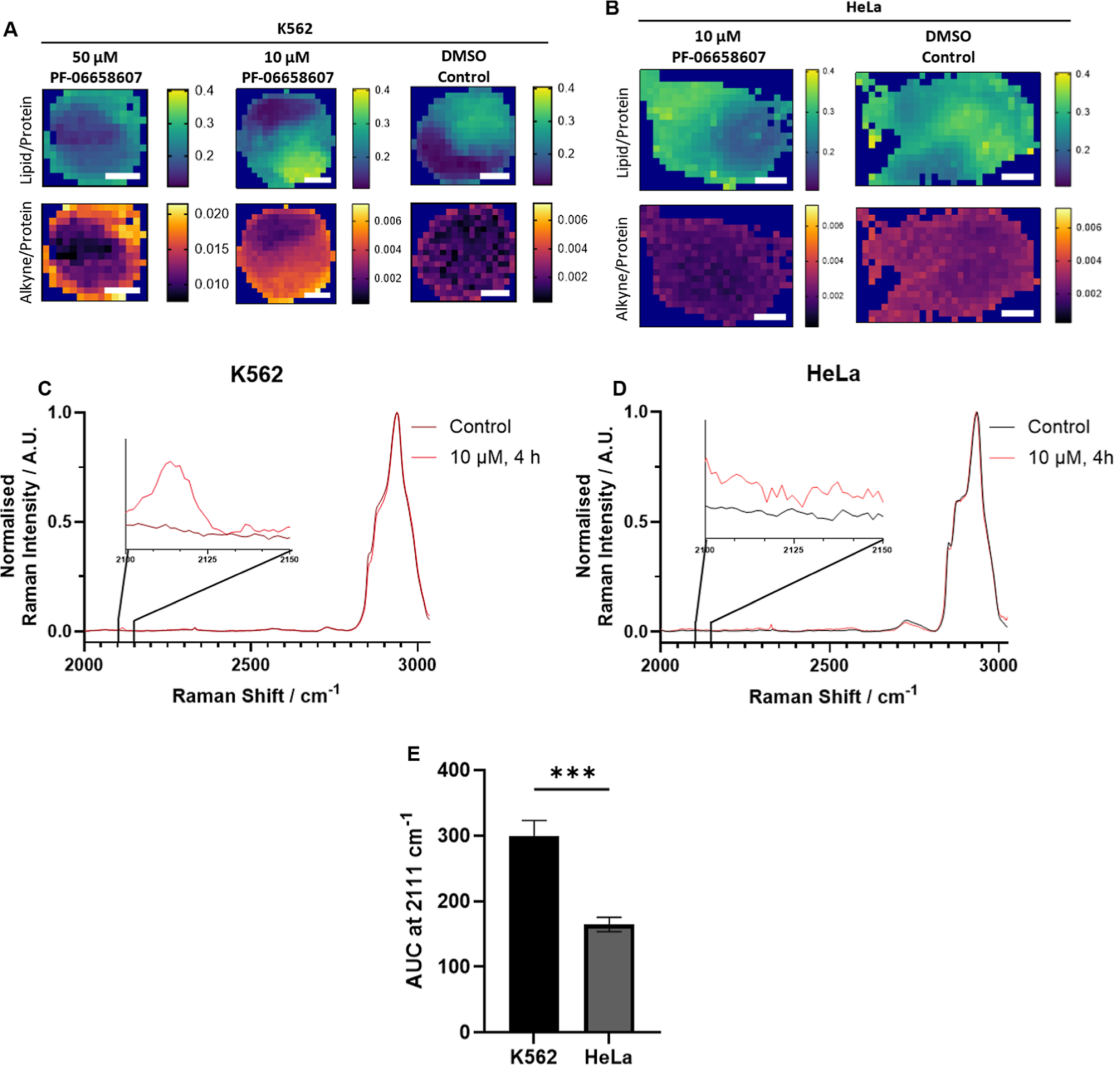
Spontaneous ratiometric Raman maps of K562 and HeLa cells. (A) K562 cells treated with ibrutinib-yne (50 μM or 10 μM, 4 h), or DMSO (10 μL, 4 h) and (B) HeLa cells treated with ibrutinib-yne (10 μM 4 h) or DMSO (10 μL, 4 h). Raman imaging utilized 532 nm excitation, 38 mW, 1 s integration time, and 1 μm step size, with scale bars: 5 μm. Mean cellular spontaneous Raman spectra of control (10 μL DMSO, 4 h) *vs* treated (ibrutinib-yne, 10 μM, 4 h); (C) K562 cells and (D) HeLa cells showing spectrum expanded view between 2100–2150 cm^–1^, with the alkyne peak around 2111 cm^–1^. (E) Area under the curve of C≡CH (2111 cm^–1^) peak from mean spectra of the Raman maps for K562 and HeLa cells. K562 cells treated with 50 μM Ibrutinib are presented with a higher ratio scale for alkyne maps to not impact visibility of those treated with 10 μM ibrutinib and to highlight a higher uptake will result in a higher alkyne intensity. AUC data represented as mean ± standard deviation (*n* = 4). An unpaired Welch’s *t*-test was performed on AUC at 2111 cm^–1^, ***: *p* ≤ 0.001.

An advantage of spontaneous Raman analysis over SRS is the greater spectral information afforded, especially in the low wavenumber region (400–1800 cm^–1^). Comparisons of the low wavenumber region between treated (10 μM ibrutnib-yne, 24 h) and control (10 μL of DMSO) K562 cells (Figure 5A) showed the effect of BTK inhibitor treatment on the spectra and therefore were able to relate this to intracellular changes. A difference spectrum between the control and treated K562 cells (Figure 5B) was also produced to exaggerate spectral differences. This highlighted an increase in the lipid signal, upon treatment, similar to what was observed using SPA, but with greater information on the profiles. An increase in the intensity of the 722 cm^–1^ Raman peak (Figure 5A,B) may indicate a larger quantity of lipids such as phosphatidylcholine and sphingomyelin, as this peak relates to the choline headgroup,^36^ and these lipids are crucial in cell signaling. These data reinforce the observation that lipid metabolism is affected in K562 cells discussed in Figure 3, as indicated by the increase in peaks around 1131, 1267, and 1450 cm^–1^ (Figure 5A,B).^36^ Peaks around 605 and 753 cm^–1^ are also increased in the treated samples, which are related to cytochrome *c*,^36^ possibly indicating some upregulation of the electron transport chain to create more energy for the cell in an attempt to counteract the inhibitive effects of the drug. This highlights the power of spontaneous Raman spectroscopy and the use of the low wavenumber region to discover drug induced effects at the cost of spatial resolution in comparison to SRS.

**Figure 5:**
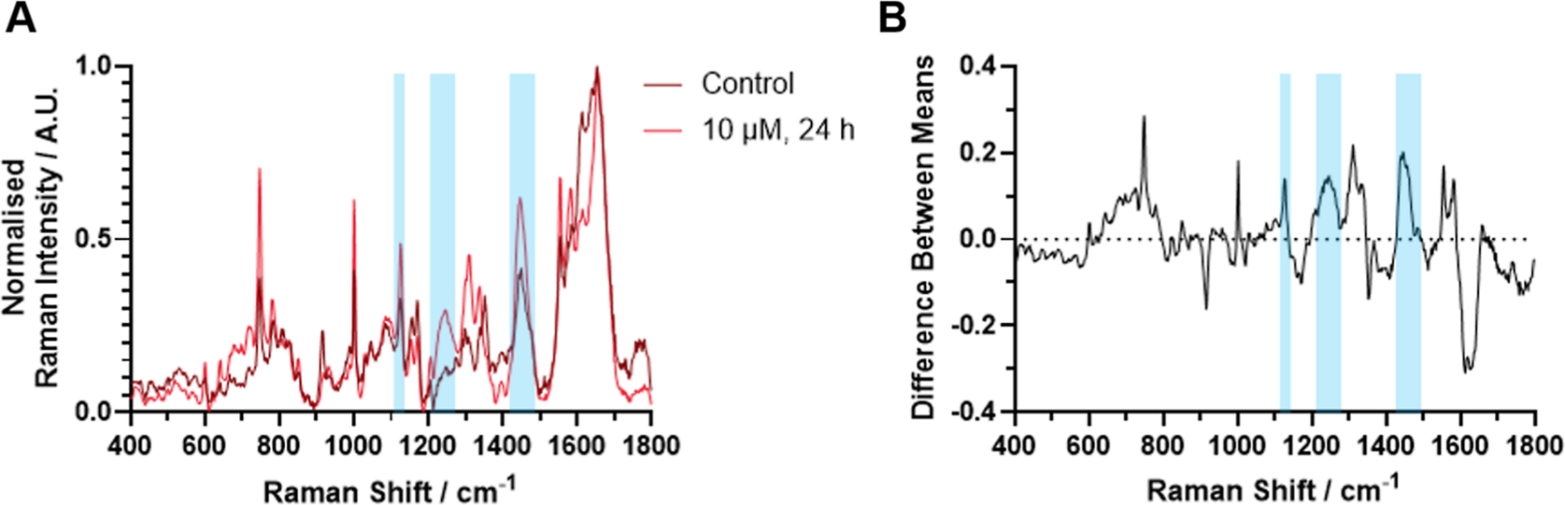
Spontaneous low wavenumber Raman spectra of (A) control (10 μL DMSO) or treated (10 μM ibrutinib-yne, 24 h) K562 cells and (B) the difference spectra of treated—control to highlight spectral differences. Spectra were acquired using 532 nm excitation, 38 mW, 1 s integration time, 10 μm step size, with a mean across the total area being taken, with peaks related to specific lipid increases upon treatment highlighted in blue.

## Conclusions

This study explored the effect of a covalently binding BTK inhibitor on selected cell lines with varying expression profiles of BTK. Initially, the expression levels of BTK in K562 and HeLa cell lines were assessed using IF to inform Raman experiments. Fluorescently tagged ibrutinib analogue, ibrutinib-FL, was found to have significant nonspecific binding, with a very high signal observed in both the high and low BTK expressing cells. The selectivity was recovered on a 24 h wash, but this greatly increased the time for a live-cell study and may have other effects. No difference in intensity was observed when using ibrutinib-yne after 30 min or 24 h washes, reinforcing the higher selectivity of this much smaller tagged drug. This shows the impact the large and bulky fluorophore on ibrutinib-FL has on the uptake, retention, and localization of the drug.

As a direct comparison for fluorescence imaging, SRS imaging of alkynyl tagged ibrutinib analogue ibrutinib-yne was performed. Superior selectivity of the drug was observed across the panel of cell lines, due to the much smaller tag required for Raman imaging imparting a reduced effect, if any, on binding to BTK, if at all, compared to ibrutinib-FL. hsSRS data was acquired to explore the distribution and identity of lipids using SPA. A significant change in LD distribution and composition upon treatment in live K562 cells was observed, with little effect in HeLa cells. This would not be possible without major perturbation of the cellular environment using fluorescence methods.

Spontaneous Raman scattering microscopy afforded much more spectral information than SRS, allowing for further probing of the distribution and endogenous lipid profiling. The distribution of ibrutinib-yne was found to be the same as with SRS, reinforcing the higher selectivity of the smaller tags compared to fluorescence. Probing the low wavenumber region of the Raman spectra allowed for a more in-depth analysis of drug-induced effect due to the greater spectral information available on intracellular contents.

This work effectively showcases the pitfalls of fluorescently tagged drugs for the use as companion imaging diagnostics due to their bulky and hydrophobic groups, which was shown to disrupt BTK inhibitor activity. By using Raman microscopy to visualize BTK inhibitor location and distribution, much smaller bio-orthogonal tags can be used to reduce off-target effects and provide a more realistic view of distribution. Making use of the rich spectral information afforded both by hsSRS and spontaneous Raman microscopy allowed the toxic responses and changes in lipid metabolism to be explored in ways not previously possible when using traditional fluorescence methods. The wide range of bio-orthogonal Raman active groups and the ability to perform label-free analysis of cellular content highlights the usefulness and versatility of Raman spectroscopy for cellular imaging and assessing drug dynamics.

## Supplementary Material



## Data Availability

The research data associated with this paper will become available from the University of Strathclyde at the following link: https://doi.org/10.15129/c9b39af6-81ea-4a2a-b5ee-fcfcb799ede4.

## Notes

Supplementary Figures S1–S3, containing IF, fluorescence and SRS images of K562 and HeLa cells showing BTK, ibrutinib-yne and ibrutinib-FL distribution (PDF)
