# Supplementary material for: Visualization of a Bruton’s Tyrosine Kinase Inhibitor Using Fluorescence and Raman Microscopy
Source: Anal Chem. 2026 May 4;98(19):13994–4003. doi: 10.1021/acs.analchem.5c04965 (PMC13191727; doi:10.1021/acs.analchem.5c04965)
Supplement: Supplementary file 1 [file ac5c04965_si_001.pdf]

Visualisation of a Bruton's Tyrosine Kinase Inhibitor using Fluorescence and Raman Microscopy

Electronic Supplementary Information

*Andrew S. Merchant, William J. Tipping, Duncan Graham, Karen Faulds**

Bionanotechnology, Department of Pure and Applied Chemistry, Technology and Innovation Centre, University of Strathclyde, 99 George Street, Glasgow, G1 1XL

*Email: karen.faulds@strath.ac.uk

Contents

Figure S1: Confocal BTK immunofluorescence (Alexa633 polyclonal mouse antibody bound to BTK monoclonal mouse antibody, red; λ_{ex} 633nm, λ_{em} 640-700 nm, co-stained with DAPI, blue; 5 µM, λ_{ex} 405 nm, λ_{em} 410-500 nm) images of **A** K562, and **B** HeLa. Scale bars are 10 µm. 3

Figure S2: Images from drug washout studies in K562 and HeLa cells. **A** fluorescence microscopy of K562 and HeLa cells treated with the fluorescent ibrutinib analogue ibrutinib-FL (500 nM, 4 h, λ_{ex} 458 nm, λ_{em} 530-630 nm), after 30 min and 24 h washes with PBS, co-stained with DAPI (5 µM, 5 min, λ_{ex} 405 nm, λ_{em} 410-500 nm), and **B** SRS images at 2930 cm^{-1} and an off-resonance subtraction of 2111 cm^{-1} (alkyne) – 2180 cm^{-1} (off-resonance) in K562 and HeLa treated with the alkynyl ibrutinib analogue, ibrutinib-yne (10 µM, 4 h) after 30 min and 24 h washes with PBS. Scale bars are 10 µm. 4

Figure S3: SRS images with increased brightness and contrast of ibrutinib-yne distribution in K562 and HeLa cells. SRS images at 2930 cm^{-1} and an off-resonance subtraction of 2111 cm^{-1} (alkyne) – 2180 cm^{-1} (off-resonance) in K562 and HeLa treated with the alkynyl ibrutinib analogue, ibrutinib-yne (10 µM, 4 h) after 30 min wash with PBS, with brightness and contrast increased to highlight a uniform background in HeLa cells. Scale bars: 10 µm...5

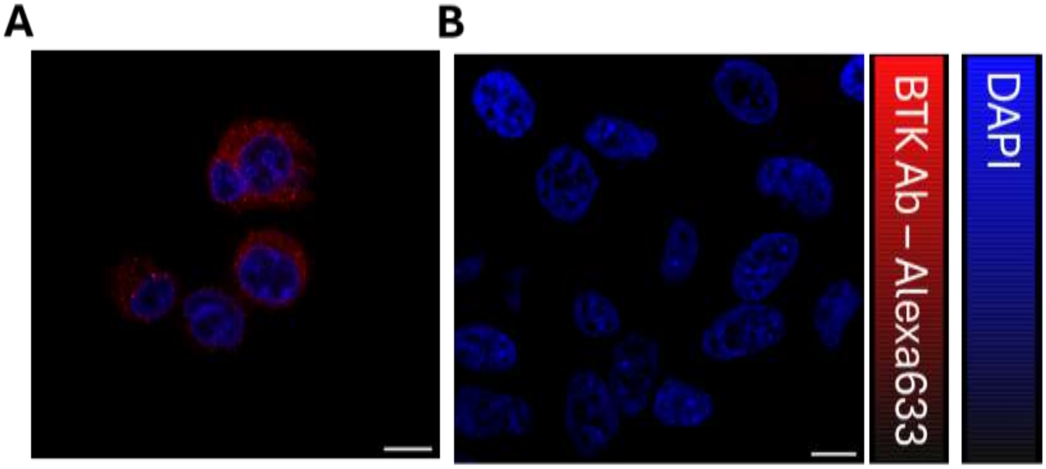

Figure S1: Confocal BTK immunofluorescence (Alexa633 polyclonal mouse antibody bound to BTK monoclonal mouse antibody, red; λ_{ex} 633nm, λ_{em} 640-700 nm, co-stained with DAPI, blue; 5 μM, λ_{ex} 405 nm, λ_{em} 410-500 nm) images of **A** K562, and **B** HeLa. Scale bars are 10 μm.

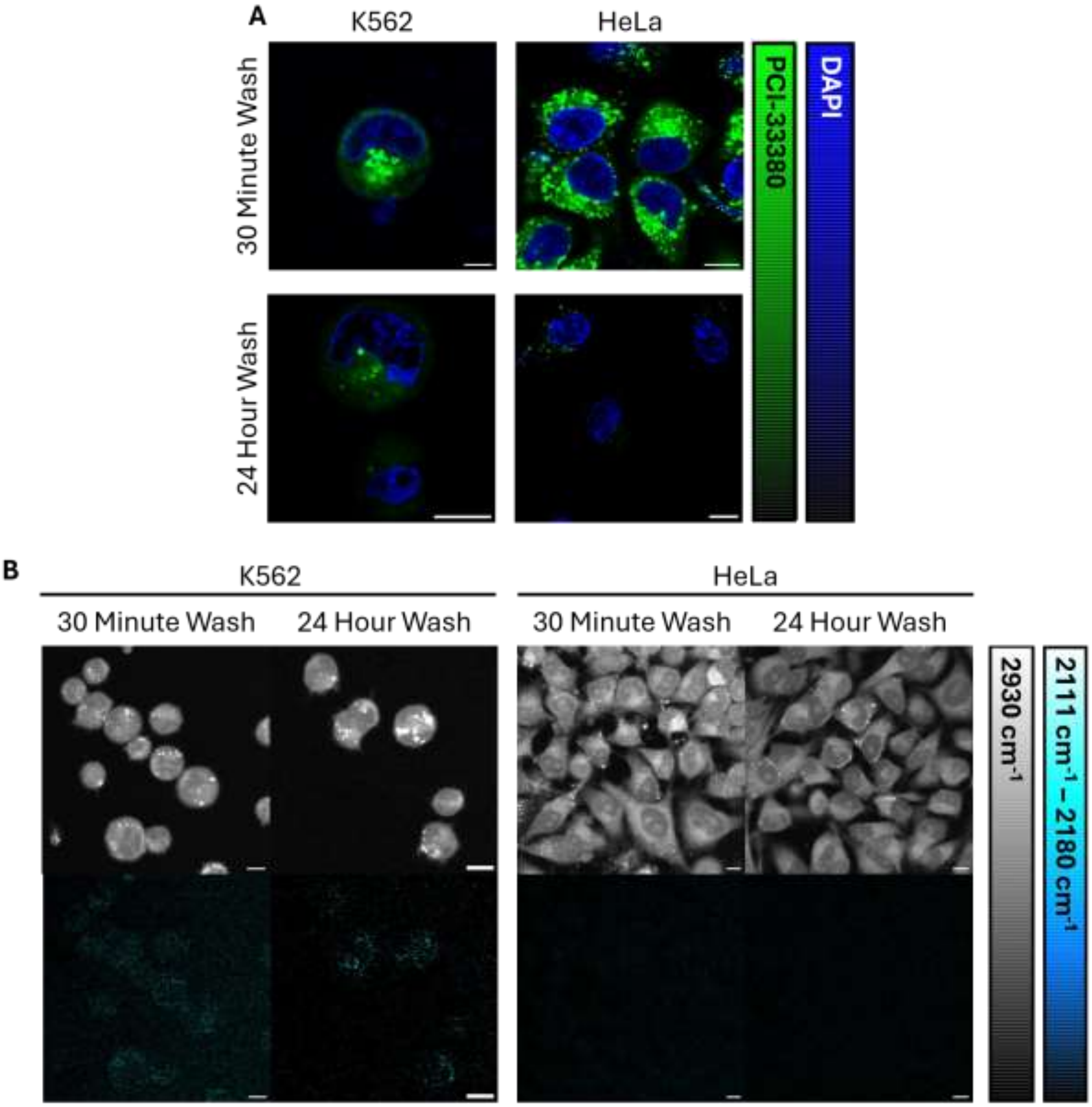

Figure S2: Images from drug washout studies in K562 and HeLa cells. **A** fluorescence microscopy of K562 and HeLa cells treated with the fluorescent ibrutinib analogue ibrutinib-FL (500 nM, 4 h, λ_{ex} 458 nm, λ_{em} 530-630 nm), after 30 min and 24 h washes with PBS, co-stained with DAPI (5 µM, 5 min, λ_{ex} 405 nm, λ_{em} 410-500 nm), and **B** SRS images at 2930 cm^{-1} and an off-resonance subtraction of 2111 cm^{-1} (alkyne) – 2180 cm^{-1} (off-resonance) in K562 and HeLa treated with the alkynyl ibrutinib analogue, ibrutinib-yne (10 µM, 4 h) after 30 min and 24 h washes with PBS. Scale bars are 10 µm.

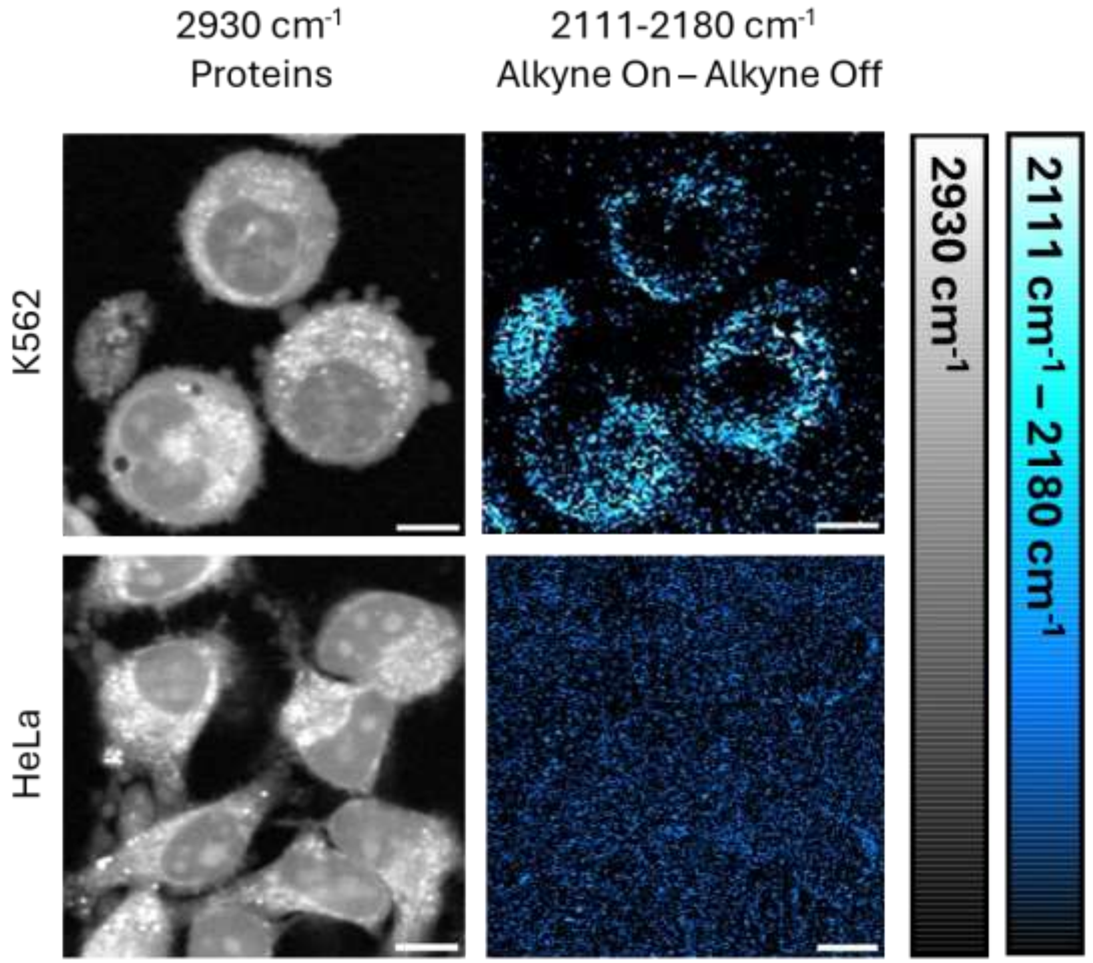

Figure S3: SRS images with increased brightness and contrast of ibrutinib-yne distribution in K562 and HeLa cells. SRS images at 2930 cm^{-1} and an off-resonance subtraction of 2111 cm^{-1} (alkyne) – 2180 cm^{-1} (off-resonance) in K562 and HeLa treated with the alkynyl ibrutinib analogue, ibrutinib-yne (10 µM, 4 h) after 30 min wash with PBS, with brightness and contrast increased to highlight a uniform background in HeLa cells. Scale bars: 10 µm
